# Soilless Cultivation: Dynamically Changing Chemical Properties and Physical Conditions of Organic Substrates Influence the Plant Phenotype of Lettuce

**DOI:** 10.3389/fpls.2020.601455

**Published:** 2021-01-18

**Authors:** Annika Nerlich, Dennis Dannehl

**Affiliations:** Division Biosystems Engineering, Faculty of Life Sciences, Albrecht Daniel Thaer-Institute of Agricultural and Horticultural Sciences, Humboldt University of Berlin, Berlin, Germany

**Keywords:** sphagnum moss, oxygen, lettuce, phenolic acids, nitrogen immobilization, rockwool substitutes, organic substrates, soilless cultivation

## Abstract

In agriculture, the increasing scarcity of arable land and the increase in extreme weather conditions has led to a large proportion of crops, especially vegetables, being cultivated in protected soilless cultivation methods to provide people with sufficient and high-quality food. Rockwool has been used for decades as a soil substitute in soilless cultivation. Since rockwool is not biodegradable, it is disposed in landfills after its use, which nowadays leads to ecological concerns and drives the search for alternative substrates, especially organic materials. The objectives of this study were to investigate the effects of organic materials (wood chips, sphagnum moss, and hemp fibers) in relation to rockwool substrate on plant growth and quality of lettuce as a result of physical and chemical properties of the mentioned substrates. We were able to show that sphagnum moss is a suitable substitute substrate for lettuce cultivation, contrary to hemp. All investigated substrates presented good physical properties, but differed in their decomposition stability. Within 8 weeks, 30% of the hemp and about 10% of both sphagnum and wood materials were degraded. It was concluded that the increased microbiological activity immobilized nitrogen and led to oxygen deficiency in the rhizosphere and resulted in increased phenolic acid contents in lettuce but poor yield on hemp. Sphagnum caused a pH decrease and accumulation of ammonium in the nutrient solution and allowed the highest yield for lettuce at moderate phenolic acid contents. Low yields were obtained on wood, which could possibly be increased by optimized nutrient solution, so that wood as an alternative to rockwool was not excluded. By applying used organic substrates as soil additives on arable land, the nutrients accumulated in it might fertilize the open field crops, thus saving mineral fertilizers. This, together with the avoidance of waste, would contribute to a greater sustainability.

## Introduction

Soilless cultivation is a modern cultivation system of plants that uses either inert organic or inorganic growing substrates, mostly in combination with nutrient solution to supply nutrients to plants. In this protected cultivation system, the yield and quality of horticultural crops can be significantly improved compared to conventional soil culture by managing the quantity and composition of the nutrient solution as well as the growing medium (Massantini et al., 1988; Xu et al., 1995). Rockwool which offers optimum physical and chemical properties has been used in greenhouse cultivation for decades (Sonneveld, 1991). An estimated area of more than 10,000 ha is cultivated in rockwool slabs worldwide, including 6,000 ha greenhouse area in Europe (Blok and Urrestarazu, 2010). The growing awareness that the disposal of rockwool substrates is not only expensive but also difficult due to their non-biodegradability and the strong negative effects on human health (Benoit and Ceustermans, 1995; Quantis, 2012) had led to the search for alternative materials. The suitability of various organic substrates as a substitute for rockwool substrates, such as wood, coconut fibers, hemp, bark, and sawdust, etc., has been investigated by many researchers (Allaire et al., 2005; Dannehl et al., 2015; Xiong et al., 2017). For a successful soilless cultivation of horticultural crops, differences in specific properties of each substrate must be considered. Various vegetable and ornamental plants have been successfully grown in bark (Wilson, 1984; Shaw et al., 2004), but also phenolic compounds with a phytotoxic effect could be extracted from the substrate (Politycka et al., 1985). Gruda (2009) noted that the activity of microorganisms should be considered when comparing potential substrates, such as bark, wood fibers, paper, and straw, to assess their suitability as growing media. Increased microbial activity can lead to nitrogen immobilization, as mineral nitrogen from the nutrient solution, is used during the mineralization of organic matter (Jansson, 1958). This reduces the nitrogen availability for plants, which in turn can lead to possible losses in product quality (Gruda, 2009). In general, several properties of a substrate, e.g., particle size, water and nutrient holding capacities, cation exchange capacity and nutrient content influence the retention, movement and availability of mineral nutrients in the root-zone (Islam et al., 2002; Sahin et al., 2002; Ao et al., 2008). Consequently, depending on the substrate properties, an adjustment of the mineral content of the applied nutrient solution should be considered in order to meet the nutrient requirements of the crops.

To produce plants in an environmentally friendly way, suitable organic substrates must be used as a substitute for rockwool. This is the only way to avoid high energy consumption and thus CO_2_ emissions during the production of rockwool. In the current study, three organic substrates were used. Wood chips were chosen because they are a waste product of wood processing. Sphagnum seems also to be a good candidate for further investigations because it was shown in a previous study by Dannehl et al. (2015) that this material has optimal physical properties for use as a growing medium. As a third option, the focus was on hemp fibers, as these give the impression of a stable and compact connection after processing. These properties could be advantageous when used as growing medium in the hydroponic system. Based on this selection, the objectives of this study were to investigate the effects of dynamically changing chemical properties and physical conditions of the mentioned organic materials in relation to rockwool substrate on plant growth and quality of lettuce. Of particular interest were physical characteristics gained from water-retention curves, substrate mineralization stability and field capacities of the substrates as well as chemical characteristics in terms of nitrogen dynamics, pH, electrical conductivity (EC) and oxygen concentrations within the different substrates.

We have chosen lettuce (*Lactuca sativa*, variety Descartes RZ) for our pot experiment because on the one hand it grows fast and on the other it is the most common salad vegetable consumed worldwide. Lettuce is known to be a good source of health-promoting compounds, especially phenolic compounds (Llorach et al., 2008; Kim et al., 2016). Usually salad is eaten raw, so that many ingredients are preserved. Substitute substrates for rockwool should not negatively influence the content of valuable ingredients and should, for example, maintain a high phenolic acid content in lettuce.

## Materials and Methods

### Experimental Set-Up and Plant Cultivation

Experiments were conducted in a Venlo-type greenhouse at Humboldt-University Berlin, Germany (Latitude 52° 46′ 74″, Longitude 13° 31′ 16″) from October 16th until December 13th 2019. Organic materials tested for their suitability as growth substrates were dried sphagnum moss, wood chips, and hemp fibers. Rockwool as an established substrate was used as a control.

Seeds of lettuce (*L. sativa*, variety Descartes RZ) were sown on October 16th in perlite and transferred to pots with different substrates on October 29th. All pots contained 52 *g* of one of the organic materials or 32 *g* of rockwool. Nine replicates per substrate were prepared and arranged in three randomized blocks. Initially all pots (*d* = 13 cm, *v* = 1 L) received 20 ml nutrient solution containing 540 mg/l Ca(NO_3_)_2_, 850 mg/l KNO_3_, 128 mg/l MgSO_4_, 180 mg KH_2_PO_4_, and 60 mg/l Fe-Chelate (Fetrilon Combi 1, Compo Expert, Münster). Further fertilization occurred successively during irrigation by supplying 76, 27, 18, 21, and 6 mg nitrate 23, 30, 37, 44, and 51 days after sowing (DAS).

As such, each pot received a total of 265 ml of nutrient solution containing 147 mg nitrate during the cultivation. Irrigation was carried out twice a week and three times a week for the last 2 weeks, recording the weights of the pots to determine the water used before replenishing them to their field capacity (FC). The average daytime temperature was set to 19°C, the average night-time temperature to 17°C during the first 33 DAS and from 34 DAS to 22°C daytime temperature and 20°C at night to accelerate growth. Additional light (63 μmol m^–2^ s^–1^) was distributed evenly over all 3 blocks from 6 am to 6 pm by high-pressure sodium lamps.

### Characterization of Growing Media Utilized for Lettuce Cultivation

#### Water-Retention Curves (pF – Curves)

The substrates to be used were first examined with regard to different physical parameters, such as total pore space (TPS), air volume (AV), bulk density (BD), and easily available water (EAW). For this purpose, metal rings with a volume of 100 cm^3^ were filled with the respective substrates, completely saturated with water and placed on a ceramic pressure plate connected to a manometer. Increasing negative pressure levels (pF-values) were used to drain different pore sizes of the previously water-saturated soil sample (pF 0). The amount of water released at each pressure stage (in our case pF 1.0 and pF 1.8) indicated the pore water volume of a defined pore size range. In this way it was possible to determine the water volume proportions [volumetric water content; θ_*V*_ (Eq. 1)] of different sizes of soil pores and thus their percentage shares in the soil. The density of water was assumed to be 1 mg cm^–3^.

(1) $$\theta_{V}\,\left[V\,o\,l\%\right]=\theta_{g}\,\left[g\,g^{-1}\right]\times \text{BD}\,\left[g\,{\text{cm}}^{-3}\right]\times 100$$

The gravimetric water content (θ*_*g*_*) is given in *g g*^–1^ and is the amount of water in g at each suction point per g substrate (Eq. 2).

(2) $$\theta_{g}\,\left[g\,g^{-1}\right]=\frac{m_{H_{2}\,O}\,\left[g\right]}{m_{\text{substrate}}\,\left[g\right]}$$

Bulk density indicates the dry mass of the substrate per 100 cm^3^ (Eq. 3).

(3) $$\text{BD}\,\left[g\,{\text{cm}}^{-3}\right]=\frac{m_{\text{substrate}}\,\left[g\right]}{100\,{\text{cm}}^{-3}}$$

According to De Boodt and Verdonck (1972) moisture content at zero suction (pf 0) is defined as TPS stated in Vol % and is the product of gravimetric water content (θ*_*g*_*) and the BD (Eq. 4).

(4) $$\text{TPS}\,\left[V\,o\,l\%\right]=\theta_{g\,\left(p\,f\,0\right)}\,\left[g\,g^{-1}\right]\times \text{BD}\,\left[g\,{\text{cm}}^{-3}\right]\times 100$$

The AV is the difference of the gravimetric water content at pF 0 and pF 1 (Eq. 5), the easy available water the difference of the gravimetric water content at pF 1 and pF 1,8 (Eq. 6).

(5) $$\text{AV}\,\left[V\,o\,l\%\right]=\theta_{g\,\left(p\,f\,0\right)}\,\left[g\,g^{-1}\right]-\theta_{g\,\left(p\,f\,1\right)}\,\left[g\,g^{-1}\right]$$

(6) $$\text{EAW}\,\left[V\,o\,l\%\right]=\theta_{g\,\left(p\,f\,1\right)}\,\left[g\,g^{-1}\right]-\theta_{g\,\left(p\,f\,1,8\right)}\,\left[g\,g^{-1}\right]$$

All physical parameters were determined in 5 replicates per substrate.

#### Field Capacity

To determine the FC, 11 pots per substrate were filled with 32 *g* rockwool, or 52 *g* of the organic substrates and saturated with water overnight. When no more water leaked from the covered pots, the difference in weight between water-filled and dry pots was used to calculate how many *g* of water per *g* of substrate was kept, representing the field capacity (Veihmeyer and Hendrickson, 1931). Means of 11 pots per substrate were calculated and used for irrigation of the plants to ensure no undesired elution of solution and nutrients from the pots. The mean pot weights at field capacity were 310 *g* for hemp, 260 *g* for wood, 530 *g* for sphagnum, and 365 *g* for rockwool. Due to the plant biomass formed or organic substrate mineralized, the target weights of the pots were adjusted to 300 *g*, 280 *g*, 550 *g*, and 385 *g* for hemp, wood, sphagnum and rockwool, respectively, on December 2nd 2019 (47 DAS).

#### Substrate Mineralization

The determination of the dry mass of all substrate pots by drying in a ventilated oven at 60°C (Heraeus, Hanau, Germany) for 7 days at the beginning and end of lettuce cultivation allowed the calculation of the mineralized organic matter during the experiment. This method was applied according to the method described by Dannehl et al. (2015). The weight reduction that occurred was expressed as percentage (%), with the root biomass not removed. The increase in weight in the rockwool substrates allowed an approximate idea of the root biomass formed in the organic substrates in which plants formed a comparable above-ground biomass as in rockwool substrates.

#### Substrate Solution Characteristics

Irrigation and fertilization of the substrates creates a “substrate solution” in the pots, which can be influenced by the organic materials used and could thus also influence the growth of the lettuce plants. The properties of the substrate solution that were studied included EC, pH-value, oxygen content and contents of ammonium, and nitrate. To obtain the substrate solution, all pots were filled with tap water to field capacity once a week and then 50 ml of tap water was added to elute excess liquid from pots. Eluates from three pots per substrate and block were collected and combined into one sample. In each sample the pH/EC value was determined according to the manufacturer’s instructions using HI9811-5 Portable pH/EC/TDS/Temperature Meter [Hanna Instruments (M), Petaling Jaya, Selangor, Malaysia] and oxygen content was determined using O_2_-Meter CG 867 (Schott Geräte GmbH, Hofheim, Germany). The substrate solution was then stored at −20°C until the ammonium and nitrate contents were analyzed with the San++ Continuous-Flow Analysator (Skalar Analytic GmbH, Erkelenz, Germany). Nitrate determination was based on a reaction with hydrazinium sulfate, sulfanilamide and N-(1-naphthyl) ethylenediamine dihydrochloride which results in the formation of a highly colored azo dye which is measured at 540 nm. The determination of ammonium was based on the chlorination of ammonium to monochloramine, which reacts with salicylate to form 5-aminosalicylate. After oxidation and oxidative coupling, a green-colored complex is formed whose absorbance is measured at 660 nm.

### Documented Plant Parameters

#### Water Usage per Plant Biomass

When the plants were watered twice a week, the weights of all pots were documented before they were refilled to field capacity to determine water consumption by evaporation and transpiration during the growing season. The yield at the harvest date and the summed-up water use was used to calculate the water use efficiency expressed as gram biomass per gram water applied.

#### Chlorophyll Contents by SPAD Meter

Chlorophyll content of leaves was nondestructively measured with a SPAD-502P portable, measuring device (Minolta Camera Co., Ltd, Osaka, Japan). The numerical SPAD value is proportional to the concentration of chlorophyll present in the leaf and is calculated from measured absorbance of a leaf in the red and near-infrared regions. Since chlorophyll content increases with the amount of nitrogen, a higher SPAD value indicates better nitrogen nutrition of the plant. Based on the plant development, readings were taken on young leaves (hemp: *n* = 3, other substrates *n* = 5) from 9 plants per substrate. SPAD values were documented starting from November 8th (23 DAS) for 6 consecutive weeks.

#### Yield, Leaf Number, Leaf Area, Dry Matter Content

Yield was determined as fresh mass per lettuce head at the end of the cultivation from 9 plants per substrate. Additionally, leaf number was counted and leaf area (LA) in cm^2^ per lettuce head was determined using a leaf area meter (Model LI 3100, LAMBDA Inst. Corp; United States) from 6 plants per substrate.

Three plants from each substrate variant and block were mixed to one sample resulting in 3 samples per substrate for further analysis. Dry mass was determined after freeze-drying (Christ Alpha 1–4, Christ; Osterrode, Germany) for 5 days. The dry matter content expressed in % was calculated by the ratio of the dry mass to the fresh mass.

#### Extraction and Determination of Phenolic Acids and Flavonoids

The freeze-dried lettuce leaves were ground to a fine powder (MM 30, Retsch GmbH, Haan, Germany) and stored at −80°C until analysis. Extraction and determination of phenolic acids and flavonoids was performed as described by Förster et al. (2015). For analysis a HPLC (Ultimate 3000, Thermo Scientific) equipped with a 150 × 2.1 mm C16 column (AcclaimPA, 3 μm, Thermo Scientific) was used. Commercially available standards of single compounds were utilized as references. The following determined response factors (RF) were used to correct the absorption differences between the internal standard 4-methoxycinnamic acid (1 mM, Sigma Aldrich; RF = 1) and the detected phenolic acids and flavonoids: RF = 1.42 for caffeoyltartaric acid (CT; Sigma-Aldrich) and di CT (Sigma-Aldrich), RF = 1.58 for caffeoylquinic acid (CQ; Sigma-Aldrich) and dicaffeoylquinic acid (diCQ; Sigma-Aldrich), RF = 2.16 for caffeoylmalic acid (CM; Phytolab), and RF = 2.15 for quercetin 3-*O*-(6″-malonylglucoside; Sigma-Aldrich).

### Statistical Methods

Data were statistically analyzed using agricolae package (de Mendiburu, 2020) in RStudio Version 1.2.5033 (R Core Team, 2020). The data were first tested for normal distribution and variance homogeneity before an analysis of variance (ANOVA) was applied and a *post hoc* analysis was performed using HSD test. Significant differences between the substrates with respect to their physical properties and influences on the substrate solution properties as well as the resulting different performances of lettuce in terms of yield and quality were calculated. Significance of statistical analyses in this research was concluded for *p* < 0.05 for a given test.

## Results

### Characteristics of Materials Used as Growing Substrates for Lettuce Cultivation

#### Physical Properties of the Different Materials Used as Substrates

In pots 52 *g* of the organic substrates and 32 *g* of the rock wool were filled up to 1 cm to the upper edge. The resulting BD per substrate and the proportion of air and water at different humidity levels in them were investigated by water-retention curves (pF curves). Results are depicted in Table 1.

**TABLE 1:** Characteristics of the air and water contents in the investigated materials used as growing substrates for lettuce cultivation.

	Hemp	Wood	Sphagnum	Rockwool	Reference*
BD (*g* cm^–3^)	0.10 ± 0.00^a^	0.10 ± 0.01^a^	0.08 ± 0.01^b^	0.05 ± 0.01^c^	<0.4
TPS (Vol %)	75.9 ± 2.9^c^	81.4 ± 2.0^b^	87.4 ± 1.6^a^	90.4 ± 2.6^a^	75–85
AV (Vol %)	13.9 ± 3.3^b^	29.6 ± 2.0^a^	12.6 ± 2.7^b^	18.9 ± 5.1^b^	10–30
EAW (Vol %)	41.4 ± 2.0^b^	22.4 ± 1.4^c^	21.0 ± 1.5^c^	70.3 ± 5.2^a^	20–30
FC (*g* H_2_O/*g*)	4.9 ± 0.1^c^	3.8 ± 0.1^*d*^	9.2 ± 0.2^b^	10.4 ± 1.0^a^	
Mineralized (%)	30.6 ± 1.0^a^	11.3 ± 1.0^b^	9.5 ± 1.5^c^	−1.7 ± 0.7	

Differences between the substrates in bulk density (BD, *n* = 5), total pore space (TPS, *n* = 5), air volume (AV, *n* = 5), easy available water (EAW, *n* = 5) Field Capacity (FD, *n* = 11), and Proportion of mineralized organic material within the 58 days of the experiment (Mineralized, *n* = 11) are indicated by different lower case letters (HSD-Test, *p* < 0.05, Mean ± Standard deviation). *Reference values for evaluation of our results were taken from the publication by Dannehl et al. (2015).

The BD of wood and hemp substrates were each 0.10 *g* cm^–3^ and thus higher than the BD of sphagnum (0.08 *g* cm^–3^) and twice as high as for rockwool (0.05 *g* cm^–3^). Sphagnum moss and rockwool had the highest TPS with 87 vol% and 90 vol%, respectively, followed by wood with a lower TPS of 81 vol% and hemp with the lowest TPS of 76 vol%. The AV was almost twice as high in wood (29.6 vol%) as in the other substrates used (Hemp 13.9 vol%, sphagnum 12.6 %, and rockwool 18.9 vol%). The proportion of EAW was highest in rockwool (70 vol%) and significantly lower in hemp (41 vol%) followed by wood and sphagnum showing the lowest share of EAW with ∼ 20 vol%. If one compares the measured parameters with the corresponding reference values, all of them are mostly within the recommended limits.

In addition to the water-retention curve, it was also investigated how much water can be stored per g substrate. The amount of water held per g substrate, depicted as field capacity (FC) in Table 1, varies significantly for each substrate. Although significantly different, Rockwool (10.4 *g* H_2_O/g) and sphagnum (9.2 *g* H_2_O/g) could hold similar amounts of water per g substrate. Hemp (4.9 *g* H_2_O/g) and wood (3.8 *g* H_2_O/g) exhibit half of the FC of rockwool and sphagnum.

Apart from the water and air balance of the substrates, their degradation stability must also be considered, as organic materials, unlike rockwool, can be mineralized during their utilization. Table 1 shows the mineralized proportion of substrate in %. 30.6% of hemp, 11.3% of wood, and 9.5% of sphagnum were mineralized during 58 days of cultivation. In the case of rockwool, an increase in the mass can be recognized by the negative sign, which is due to the roots of the lettuce plants growing into the substrate. It should be noted that the roots could not be removed from the substrates at the end of the experiment, because they were to firmly anchored in the substrates.

#### Influence of Different Substrates on pH, Electric Conductivity, Oxygen Concentration and Concentrations of Ammonium and Nitrate Within Substrate Solution

Several properties of the nutrient solution can influence the yield and/or quality of lettuce, including EC, pH-value, oxygen concentration of the solution and the concentration of nutrients or chemical forms of the elements. Since it was expected that the different substrates would change the properties of the nutrient solution added to the substrate pots, part of the solution contained in the pots (substrate solution) was eluted and analyzed weekly for 6 consecutive weeks.

**FIGURE 1:**
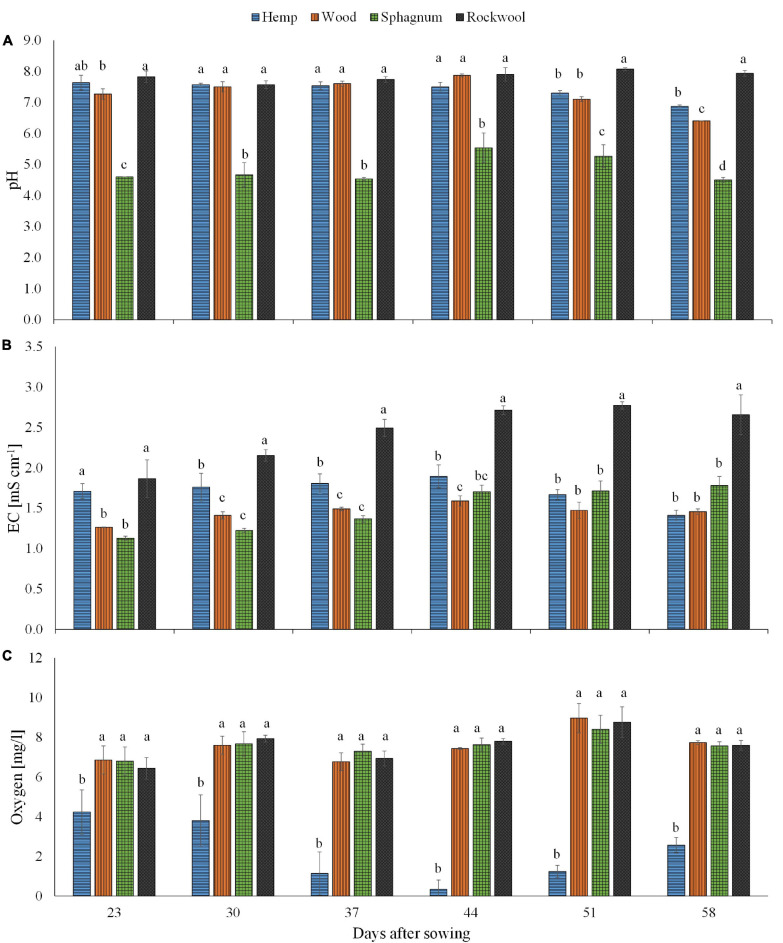
Measured pH **(A)**, EC **(B)**, and oxygen concentration **(C)** in substrate solution eluted from pots filled with different substrates for cultivation of lettuce. Measurements were taken during 6 consecutive weeks until harvest. Significant differences between the substrates are indicated by different lower case letters (HSD-Test, *p* < 0.05, Mean ± Standard deviation, and *n* = 3) EC: electrical conductivity.

Looking at the pH values in Figure 1A, it is noticeable that at all times the pH values of the sphagnum substrate solution were the lowest and ranged between 4.5 and 5.5, whereas the pH values of the rock wool substrate solution were the highest at all times and ranged between 7.6 and 8.1. At the time points 30, 37, and 44 days after sowing, there was no significant difference in the pH value of the substrate solution between rockwool, hemp and wood. In the last 2 weeks, the pH value in the substrate solution of hemp and wood decreased significantly compared to rockwool and in the last week in substrate solution of wood more strongly than in hemp.

Regarding the EC values in Figure 1B, it is striking that this was highest for rockwool at all times and seemed to increase over time, starting from 1.87 mS cm^–1^ and finish at 2.66 mS cm^–1^. At the beginning, the EC in hemp substrate solution (1.71 mS cm^–1^) was similar to that in rockwool, but in the following weeks it was always significantly lower than in rockwool, ranging between 1.41 mS cm^–1^ and 1.89 mS cm^–1^. During the first 3 weeks, the lowest EC values were always in substrate solutions of wood and sphagnum with values between 1.13 mS cm^–1^ and 1.49 mS cm^–1^. In the following weeks EC of hemp, wood and sphagnum substrate solutions leveled off to a similar degree.

The oxygen concentration in substrate solutions of hemp was lowest at all measured time points, starting at 4.2 mg/l and declining to almost zero at 44 days after sowing (DAS) before increasing again to 2.6 mg/l at the last date (Figure 1C). Substrate solutions from the other substrates contained similar oxygen concentrations, but significantly higher oxygen concentrations than hemp solution at all dates, ranging between 6.4 and 9.0 mg/l (Figure 1C).

**FIGURE 2:**
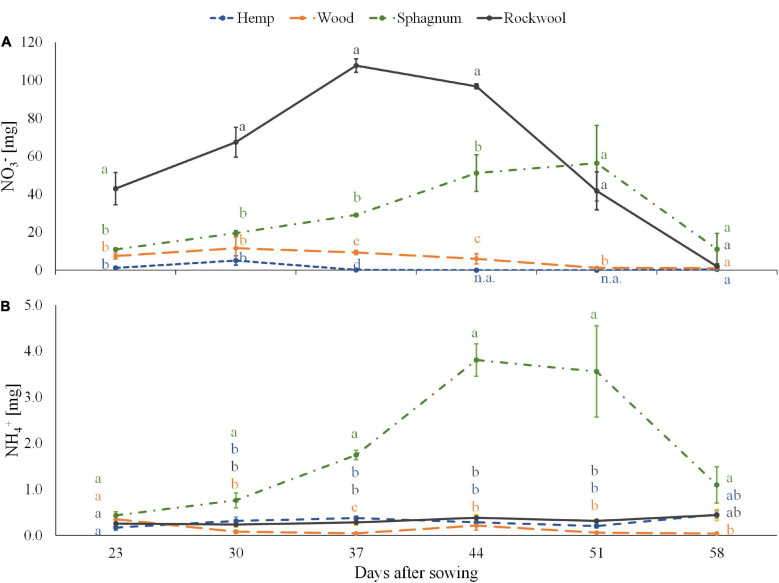
Ammonium **(A)** and nitrate **(B)** contents in substrate solution eluted from pots filled with different substrates for cultivation of lettuce. Measurements were taken during 6 consecutive weeks until harvest. Significant differences between the substrates are indicated by different lower case letters (HSD-Test, *p* < 0.05, Mean ± Standard deviation, and *n* = 3) n.a.: not available (below detection limit).

Equal amount of nitrogen was added successively to each pot during the experiment in the form of nitrate. Because we used organic materials as substrate, the substrate solution could contain released ammonium from the organic substance in addition to the fertilized nitrate. Therefore, the amounts of ammonium and nitrate per pot were determined in 6 consecutive weeks (Figure 2). The amount of nitrate per pot was highest in the rockwool variety and corresponded to the weekly nutrient doses, which first increased up to 108 mg nitrate per pot till the 3rd week and then decreased to 2 mg nitrate per pot in the last week. In Sphagnum solutions lower amounts of nitrate than in rockwool solutions were measured during the first 4 weeks peaking at the 5th week at a similar amount of nitrate (56 mg) as in rockwool solutions. Lower amounts in nitrate were found in pots of wood from the 3rd week onward constant decreasing from 11.5 mg per pot to 1 mg at the end. Nitrate in hemp samples was below the lower detection limit almost at every date analyzed. At the final monitoring point, all substrate solutions contained similarly low nitrate contents.

All pots contained equal low amounts of ammonium at the start of the measurements, between 0.17 and 0.44 mg per pot. While wood, hemp and rockwool maintained similarly low levels of ammonium per pot throughout the 6 weeks, in sphagnum the ammonium content increased continuously from the second week and reached its highest level of 3.81 mg at 44 DAS before starting to decrease.

### Influence of Different Growth Substrates on Plant Growth, Yield and Quality

Early on in the experiment, it became apparent that plant growth in the different substrates was clearly different. On the one hand by the size and number of leaves of the lettuce (data not shown) and on the other hand by the different green coloring of the leaves. Results of documented plant parameters are described in detail in the following sections.

#### Influence of Different Growth Substrates on Water Use Efficiency

**FIGURE 3:**
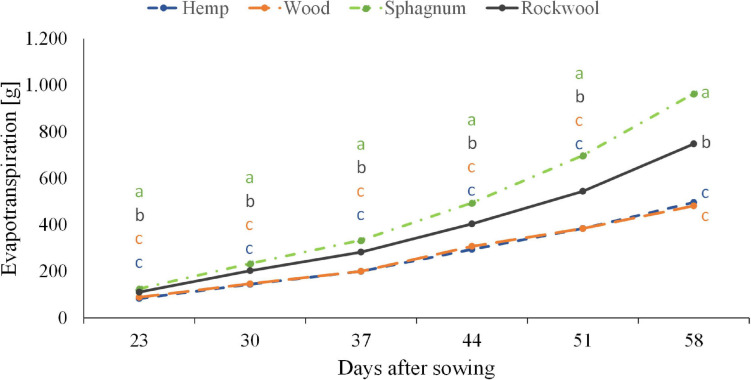
Influence of different substrates on evapotranspiration of lettuce plants grown in pots. Measurements were taken twice a week. Significant differences between the substrates are indicated by different lower case letters (HSD-Test, *p* < 0.05, Mean ± Standard deviation, and *n* = 9).

When lettuce was grown on different substrates, water usage differed significantly among variants (Figure 3). From the beginning till the end, plants grown on sphagnum used most water followed by rockwool grown plants. Hemp and wood grown plants used similar amounts of water, but significantly less than those from sphagnum and rockwool. Finally, plants grown on sphagnum were irrigated with 965 *g*, on rockwool with 748 *g*, on wood with 482 *g*, and on hemp with 496 *g* water. As the plants exhibited different growth on the substrates, it was useful to compare the biomass produced per given amount of water, as this is the productive part of the water consumption in contrast to the evaporation from the substrates. As can be seen in Table 2, the plants on rockwool and sphagnum were most effective in forming fresh mass with 23.24 *g* FM and 25.45 *g* FM per *g* of water, respectively. Wood grown plants could synthesize 6.36 *g* FM per g water, yielding just a quarter of biomass compared to sphagnum. In hemp substrates, plants were extremely retarded in growth and just reached 0.39 *g* FM per g water.

**TABLE 2:** Effects of different growth substrates on leaf number, leaf area, yield, dry matter content, and fresh mass (FM) per *g* irrigated water of lettuce heads.

	Hemp	Wood	Sphagnum	Rockwool
Leaf number	7 ± 1^c^	24 ± 2^b^	54 ± 4^*a*^	50 ± 3^a^
Leaf Area (cm^2^)	7 ± 2^*d*^	175 ± 23^c^	873 ± 160^a^	692 ± 53^b^
Yield (*g* FW)	0.2 ± 0.1^*d*^	3.1 ± 1.0^c^	24.6 ± 3.6^a^	17.2 ± 1.9^b^
DM content (%)	17.1 ± 3.2^a^	8.8 ± 0.5^b^	5.4 ± 0.1^b^	5.4 ± 0.3^b^
FM (g)/H_2_O (g)	0.39 ± 0.11^c^	6.36 ± 2.17^b^	25.45 ± 2.94^a^	23.24 ± 3.20^a^

*Significant differences between the substrates are indicated by different lower case letters [HSD-Test, *p* < 0.05, Mean ± Standard deviation, *n* = 9, for DM content *n* = 3, for FM (*g*) H_2_O (g) ^–1^: *n* = 6].*

#### Influence of Different Growth Substrates on Chlorophyll Content in Lettuce

**FIGURE 4:**
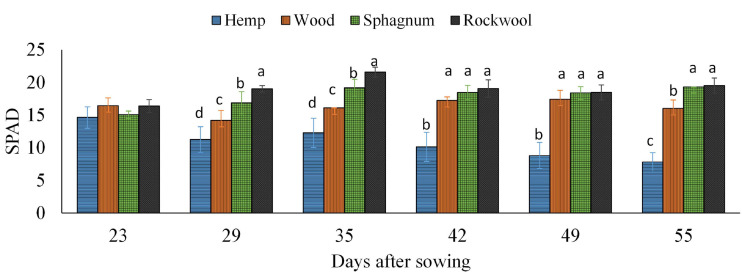
Measured SPAD values in lettuce plants grown in different substrates. Mean SPAD values measured on 6 consecutive weeks. Significant differences between the substrates are indicated by different lower case letters (HSD-Test, *p* < 0.05, Mean ± Standard deviation, and *n* = 9).

To confirm the impression of the previously mentioned observed different green colorations, the SPAD values of all plants were recorded weekly and are depicted in Figure 4. During the experiment, the lowest SPAD values were measured in leaves of hemp grown plants at each measurement time, which also decreased continuously over time from 14.6 to 7.8. In contrast, the SPAD values of lettuce leaves grown on rockwool were always highest and ranged from 16.4 to 21.6. The SPAD values of lettuce grown on wood or sphagnum did not significantly differ from SPAD values of the rockwool variety, except at 29 and 35 DAS. At 29 and 35 DAS, SPAD values were increasing in the order hemp < wood < sphagnum < rockwool.

#### Influence of Different Growth Substrates on Yield

Hemp plants were strongly reduced in growth, exhibiting the lowest leaf number (7), leaf area (7 cm^2^) and yield (0.2 *g* FW) and the highest DM content (17.1%) from all varieties (Table 2). Lettuce plants from wood performed slightly better than plants from hemp with 24 leaves, 175 cm^2^ leaf area and 3.1 *g* yield. Leaf number of plants from rockwool (50) and sphagnum (54) were similar and highest, but significantly different in leaf area (rockwool: 692 cm^2^; sphagnum: 873 cm^2^) and thus yield (rockwool: 17.2 *g*; sphagnum: 24.6 *g*). The dry matter content of plants grown on wood (8.8%) seems to be higher than that of plants grown on rockwool (5.4%) and sphagnum (5.4%), but this was not significant different.

#### Influence of Different Growth Substrates on Secondary Metabolites in Lettuce

As nutritionally valuable ingredients, secondary metabolites such as phenolic acids and flavonoids are important for the quality of lettuce. We were able to detect the following phenolic acids in lettuce: caffeoyltartaric acid (CT), caffeoylquinic acid (CQ), caffeoylmalic acid (CM), dicaffeoyltartaric acid (diCT), and diCQ. Quercetin 3-O-(600-malonylglucoside; QM-glucoside) was also found as representative of flavonoids (Table 3). Total flavonoid concentration was highest in hemp leaves (0.50 mg/*g* DM) followed by wood leaves (0.18 mg/*g* DM). In plants from rockwool (0.05 mg/*g* DM) and sphagnum (0.06 mg/*g* DM) comparable low concentration of flavonoids were found. A similar result was found for total phenolic acids, which concentration were much higher than concentrations of flavonoids. Plants grown on hemp and wood substrates had concentrations of about 20 mg, twice as high as plants grown on sphagnum or rockwool. The most abundant phenolic acid was diCT. In comparison within the substrate variants, the concentration of diCT is highest in plants cultivated on hemp and wood and lowest in plants of rockwool and sphagnum. DiCQ was the least detectable compared to the other phenolic acids considered and was significantly higher in lettuce grown on hemp than in plants of all other substrates. Concentration of CQ was highest in plants from the hemp variant, followed by wood. Least concentrations of CQ were found in plants from rockwool and sphagnum. Regarding CT, concentrations ranged from 2.17 mg/*g* DM in wood, to 1.24 mg/*g* DM in Hemp and almost 1.7 mg/*g* DM in plants from sphagnum and rockwool. Concentrations of CM was the lowest in plants from hemp and sphagnum, higher in plants from wood and highest in plants from rockwool.

**TABLE 3:** Concentrations of phenolic acids and flavonoids in lettuce caused by different substrates.

	Hemp	Wood	Sphagnum	Rockwool
	**Phenolic acids (mg/*g* DM)**			
Caffeoyltartaric acid	1.24 ± 0.17^c^	2.17 ± 0.05^a^	1.68 ± 0.06^b^	1.66 ± 0.14^b^
Caffeoylquinic acid	6.17 ± 0.27^a^	4.23 ± 0.22^b^	1.50 ± 0.15^c^	1.06 ± 0.14^c^
Caffeoylmalic acid	0.45 ± 0.06^c^	0.71 ± 0.01^b^	0.65 ± 0.10^*bc*^	0.93 ± 0.07^a^
Dicaffeoyltartaric acid	10.69 ± 0.79^a^	12.59 ± 0.56^a^	6.69 ± 0.49^b^	5.97 ± 0.60^b^
Dicaffeoylquinic acid	2.63 ± 0.17^a^	0.19 ± 0.05^b^	0.19 ± 0.08^b^	0.10 ± 0.03^b^
Total phenolic acids Σ	21.18 ± 0.96^a^	19.89 ± 0.52^a^	10.72 ± 0.60^b^	9.72 ± 0.97^b^

	**Flavonoids (mg/*g* DM)**			
Quercetin 3-*O*-(6″-malonylglucoside)	0.50 ± 0.05^a^	0.18 ± 0.03^b^	0.06 ± 0.00^c^	0.05 ± 0.00^c^

*Significant differences between the substrates are indicated by different lower case letters (HSD-Test, *p* < 0.05, Mean ± Standard deviation, and *n* = 3).*

**FIGURE 5:**
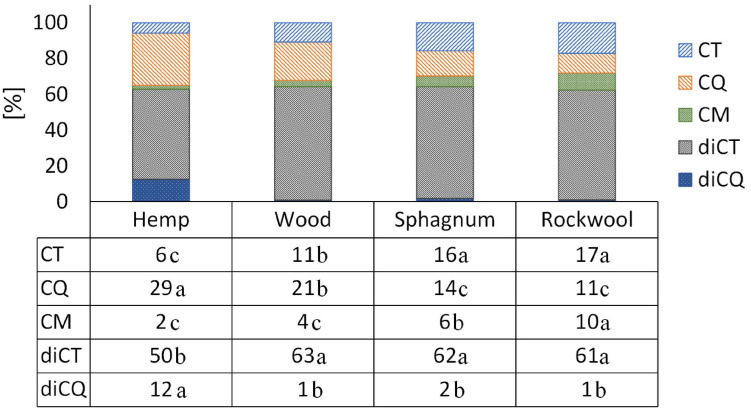
The individual phenolic compounds as a proportion of the total amount of phenolic acids in the lettuce. Significant differences between the substrates are indicated by different lower case letters (HSD-Test, *p* < 0.05, Mean ± Standard deviation, and *n* = 3); CT: caffeoyltartaric acid, CQ: caffeoylquinic acid, CM: caffeoylmalic acid, diCT: dicaffeoyltartaric acid, and diCQ: dicaffeoylquinic acid.

Comparing the proportion of individual phenolic acids, a clear shift is visible in plants grown on hemp from diCT, CT, and CM toward diCQ and CQ when comparing the phenolic acid profiles with plants from rockwool or sphagnum (Figure 5). The phenolic acid profile in plants from wood showed a similar proportion of diCT and diCQ, but a shift from CT and CM toward CQ in comparison to rockwool and sphagnum. The proportions of the different phenolic acids were similar in plants of sphagnum and rockwool, except for CM which accounted for a lower proportion of phenolic acids in sphagnum.

## Discussion

Organic materials, to use as substitutes for the established rockwool in soilless cultivation, should enable the producer to achieve optimal yields and quality of the cultivated crops. In the ideal case, organic materials can be obtained as a waste product from other production processes, e.g., wood chips from the wood processing industry. If these would be amended on arable land after use as a substrate in hydroponic cultivation, this could help closing nutrient cycles and avoiding waste. In the present study, we tested hemp fiber, wood chips, and sphagnum moss as alternative substrates for rockwool. All substrates were examined for different physico-chemical properties and their effects on lettuce growth and quality.

Firstly, we checked physical properties like BD, TPS, AV, and EAW to see whether chosen organic materials might be suitable as substrates. The reference values for the evaluation of our results of water retention measurements were taken from the publication by Dannehl et al. (2015), which summarizes reference values from various sources. A comparison between the measured values and those recommended from other studies for BD, TPS, AV, and EAW showed that the substrates we investigated are almost all within the reference values, even if significant differences were found between the substrates (Table 1). Bulk densities ranged from 0.05 *g* cm^–3^ (rockwool), 0.08 *g* cm^–3^ (sphagnum) to 0.10 *g* cm^–3^ (hemp and wood), which are similar to those found by Dannehl et al. (2015). The TPS in the current study was largest in rockwool (90 vol%) and sphagnum (87 vol%) followed by wood (81 vol%) and hemp (76 vol%). This contradicts the results published by Dannehl et al. (2015), but is in a similar range, which is not true for the AV and EAW values. The air-filled pores have a proportion of 13 vol% in sphagnum, 14 vol% in hemp, 19 vol% in rockwool. The highest proportion of air-filled pores is 30 vol% in wood. Again, all substrates are within the reference range (10–30 vol%). The proportion of EAW in hemp (41 vol%) and rockwool (70 vol%) is well above the reference range of 20–30 vol% (De Boodt and Verdonck, 1972), whereas wood and sphagnum are within this range. In Gruda and Schnitzler (2004) described values for EAV and AV of wood chips were in a similar range in our study, but we measured a lower TPS. Nevertheless, these results indicate that the selected organic materials could be suitable as substrates. As an additional factor, the mineralization in which the organic materials differ from the stable rockwool was also examined in Table 1. Since hemp had decomposed already to 30% within the relatively short time of the experiment of 53 days, it should be considered whether this substrate could be used in hydroponic greenhouse cultivation, if the cultivation period for, e.g., tomatoes is significantly longer. In this case, wood and sphagnum substrates with a lower degradation of ∼11% in 53 days would be more suitable. It should be noted that the roots remained in the substrate at the end. This resulted in higher masses of organic material being detected than were actually present, which causes the mineralization to be somewhat underestimated. The mineral rockwool is not degraded, but shows a mass increase of 1.7% at the end of the experiment, which results from the ingrowing roots.

The differences shown in the degradability of the substrates can have an impact on the physico-chemical properties during the entire crop cycle as was shown for wood fiber and coconut fiber in Domeño et al. (2009). Different organic materials might influence the composition of mineralizing microorganisms and thus microclimatic conditions created and/or substances synthesized by the activity of microorganisms during mineralization. It was known for sphagnum to facilitate lower pH levels (Clymo, 1967) in surrounding water, so in our case within the substrate solution. Processes influenced by pH include nutrients solubility and availability as well as microorganism activity. The measured pH values in peat moss eluates were in the range of 4.5 to 5.5 which is lower than the pH proposed pH for lettuce cultivation (5.9; Knight and Mitchell, 1983), whereas the pH values of the other substrate eluates were higher than 7 and for rockwool almost 8, except for the last week (Figure 1A). In wood, hemp and rockwool substrates the pH measured was higher than recommended which opens up the possibility that nutrients are no longer as readily available as at lower pH levels and may have led to the poorer yields compared to sphagnum. The lower pH values in the substrate solution of sphagnum can be traced back to unesterified polyuronic acids, which can account for 10–25% of the dry weight of sphagnum, depending on the species (Clymo, 1964).

The EC was highest in rockwool at all time points, ranging from 1.9 mSi cm^–1^ to 2.8 mSi cm^–1^ and seems to reflect the fertilization regime during cultivation (Figure 1B). Increasing EC values over time in rockwool substrates resulted from evaporated water from the pots, leaving nutrient salts in the solution. Our measured EC values in rockwool eluates are in the recommended range of 2.0–3.0 mSi cm^–1^ according to Economakis (1991), who also showed in his study on two lettuce varieties that their fresh mass was hardly affected by changes in EC values within a range of 1.5–5.0 mSi cm^–1^. The lower EC (1.3–1.9 mSi cm^–1^) values of organic substrate eluates at all time points might indicate nutrients to have been bound to the substrate or to have been incorporated into microbial biomass.

It was initially assumed that the microbial biomass that developed during the test period consumed oxygen from the substrate solution and thus could lead to oxygen deficiency in the rhizosphere of the plants. The assumption was supported by an odor of decay, which was perceived rising from the hemp substrates, indicating anaerobic degradation processes. As shown in Figure 1C, a decrease in oxygen was detected in hemp substrate eluates, exclusively (Figure 1C), suggesting higher activity of microorganisms than in other substrates tested. These result fits well with the result of the substrate stability of hemp, which was lowest in our experiment (Table 1) and to data of nitrate contents in pots (Figure 2A). The amounts of nitrate in the eluate from rockwool substrates corresponded to the weekly nutrient doses added to the pots and prove that more than sufficient nitrate was available for yield formation. In the sphagnum variety nitrate might be incorporated into microbial biomass as well as in plant biomass, resulting in lower amounts of nitrate in the eluate when compared to rockwool. Nitrate in hemp solution was lowest, close to the lower detection limit, and at 44 and 51 DAS not detectable, although all substrate pots received equal amounts of nutrients. It is highly probable that nitrate was used to build up microbial biomass, thus reducing the availability of nitrate for plants, which led to strongly retarded growth in the hemp variant. Lettuce plants grown on wood exhibited better growth than lettuce plants from hemp. The reason for improved growth might be the higher mineralization stability of wood in comparison to hemp, resulting in lower microbial activity and thus in higher oxygen and nitrate levels within the root zone. Sphagnum was similarly susceptible to degradation as wood, contained similar amounts of oxygen and had similar EC values as wood, but supported plant growth best. Beside the afore mentioned higher nutrient availability due to the lower pH in Sphagnum, the high contents of ammonium in sphagnum pots (Figure 2B) might support a better growth of lettuce. The sphagnum variant was the only one to show significant contents of both N-forms in its substrate solution. A suitable balance of nitrate and ammonium nutrition compared to the use of either nitrate or ammonium sources alone has led to an increase in vegetable yield and nutritional quality in many vegetable crops, e.g., tomato (Claussen, 2002), spinach (Wang et al., 2009), endive (Santamaria and Elia, 1997), and lettuce (McCall and Willumsen, 1998). In rockwool substrates, ammonium was almost absent (Figure 2B) and nitrate was highest (Figure 2B) during cultivation, which, in combination with highest EC values detected, might be a reason for less yield compared to the yield from sphagnum substrates. Nutrition with different forms of nitrogen also has an effect on the pH value of the nutrient solution. It is known that a nitrate nutrition leads to increased pH values due to OH^–^ or HCO_3_^–^ released into nutrient solution to balance ion uptake by the plant roots, whereas ammonium nutrition leads to a lowering of the pH values because of H^+^ excretion during NH_4_^+^ uptake and nitrification of NH_4_^+^-N to NO_3_^–^-N within the solution which releases H^+^ as well (Barker and Mills, 1980). Since we fertilized the plants with NO_3_^–^-N and detected NH_4_^+^-N in sphagnum solution exclusively, this could contribute additionally to the observed differences in pH beside the mentioned unesterified polyuronic acids in sphagnum substrate. To what extent there is a connection between the low pH value in sphagnum substrate and the presence of ammonium is unclear. A shift in pH toward acidity can lead to a shift in the microbial decomposer community from bacteria to fungi (Rousk et al., 2009). Sphagnan present in sphagnum, also was shown to influence microorganisms. The pectin-like polysaccharide is considered for antibacterial and wound healing effects (Børsheim et al., 2001). Differences in the opacity of the eluates obtained from the substrates (hemp very opaque, other substrate eluates clear; data not shown) indicate different compositions and quantities of mineralizing organisms. To what extent this is responsible for the accumulation of ammonium in sphagnum solution remains the subject of further studies. Different compositions of the microbial population in the wood and sphagnum substrates could mean different nitrogen requirements for the formation of microbial biomass and its metabolites. This could also explain why, at similar mineralization stability, differences in the amount of nitrate were found in the eluates. The different contents of the two N-forms per pot influences the N-nutritional status of the plant. Nitrogen within the plant is needed for chlorophyll synthesis. Chlorophyll concentrations in leaves were measured indirectly by SPAD meter. The SPAD values obtained in turn allow conclusions to be drawn about the nitrogen supply status of the plant (Blackmer and Schepers, 1995). During growth period, clear differences in SPAD values were found 29 and 35 DAS with increasing values from hemp > wood > rockwool > sphagnum. No differences in SPAD between wood, sphagnum and rockwool were present in the last 3 weeks. Plants from hemp had the lowest SPAD values at all time points, reflecting low nitrate/ammonium contents in substrate solution (Figure 2). Nitrate was high in rockwool and sphagnum at 44 and 51 DAS, while ammonium was high just in sphagnum substrate solution at these time points. SPAD Values around these time points gave similar values not just for sphagnum and rockwool grown plants, but for wood grown plants as well. Substrate solution from wood contained much lower contents of any of the N-forms analyzed than from sphagnum or rockwool. It can be assumed that the lettuce on wood meets the lower supply of nitrogen with limited growth in order to keep the nitrogen concentration in the plant at a constant level or allocated more biomass to roots for enhanced nitrogen uptake what was shown for example for 27 herbaceous plants by (Müller et al., 2000).

The different growth is confirmed by the data for yield, leaf number and leaf area in Table 2. While the leaf number of lettuce plants from sphagnum and rockwool do not differ significantly, the leaf area is largest in plants grown on sphagnum and so is the yield. Yields and leaf areas decreased in the order sphagnum > rockwool > wood > hemp. Due to the different growth in the variants, water used per gram of biomass created differed significantly (Table 2). Most biomass per g water given was created on sphagnum and rockwool. Only one third of the biomass formed on sphagnum or rockwool was formed on wood and almost no biomass was formed on hemp.

As the focus is increasingly being placed on product quality in addition to yield, we have looked at the phenolic acid and flavonoid content of the leaves, as these are considered to be nutritionally valuable components due to their health-promoting properties (Birt et al., 2001; Nicolle et al., 2004). The production of phenolic compounds in plants is strongly influenced by nitrogen availability, whereby a reduction in nitrogen availability generally leads to an increase in phenolic acid concentrations. For lettuce this has already been shown in studies by Qadir et al. (2017) and Zhou et al. (2018), but is true for other species as well, e.g., artichoke and cardoon (Rouphael et al., 2012). This could also be shown in our results (Table 3 and Figure 2). Nitrogen was found in pots of hemp and wood in the smallest amounts and led to twice as high total phenolic acid concentrations in the leaves as in the plants on rockwool and sphagnum. The concentration of the flavonoid Quercetin 3-*O*-(6″-malonylglucoside) in hemp and wood grown plants was even several times higher than the concentrations in leaves of rockwool or sphagnum varieties. The different substrates not only caused different concentrations in the phenolic acids and flavonoids, but also a shift in the proportions of the individual phenolic acids within the total phenolic acids. The phenolic acid pattern in lettuce heads of hemp substrates was shifted toward CQ derivatives at the expense of CT derivatives compared to the phenolic acid profiles of lettuce from rockwool (Figure 5). Also in the wood variant, the phenolic acids are shifted toward CQ at the expense of CT and CM. Cultivation on sphagnum led to no change in the phenolic acid pattern compared to cultivation on rockwool. A study by Zhou et al. (2019) showed an increase in the proportion of a CQ derivative in lettuce under nitrogen-limiting conditions as well and is therefore consistent with our results. In nutrient solution aeration experiments with hydroponically cultivated lettuce Encarnación et al. (2015) found higher phenolic acid levels in lettuce plants in non-aerated nutrient solutions than in lettuce from aerated nutrient solutions. Since similar higher phenolic acid concentrations in lettuce from hemp and wood were measured in our experiments, but the severe oxygen deficiency only occurred in hemp, the availability of nitrogen seems to have a greater effect on phenolic acid concentrations.

## Conclusion

Even though the highest phenolic acid and flavonoid content in lettuce has been found on hemp substrates, the use of hemp as a substrate is not advisable due to the extremely low yield and the high instability. Yields caused by wood substrates were higher, but did not reach the yields of rockwool and sphagnum. Nevertheless, wood could be a suitable alternative to rockwool if the nutrients in the nutrient solution, which are removed by immobilization, would be optimized. The extent to which yields on wood substrates can be increased by controlling pH and nutrient levels, while maintaining increased phenolic acid and flavonoid levels, needs to be investigated in more detail. In our study, sphagnum was found to provide optimal properties for lettuce growth and is a good substitute for rock wool.

## Data Availability Statement

The raw data supporting the conclusions of this article will be made available by the authors, without undue reservation.

## Author Contributions

AN and DD contribute equally to the study conception and design, acquisition, analysis and interpretation of data, and drafting of the manuscript. Both authors contributed to the article and approved the submitted version.

## Conflict of Interest

The authors declare that the research was conducted in the absence of any commercial or financial relationships that could be construed as a potential conflict of interest. The handling editor declared a shared affiliation, with the authors, at the time of the review.

## References

[B1] Allaire S. E. Caron J. Ménard C. Dorais M. (2005). Potential replacements for rockwool as growing substrate for greenhouse tomato. *Can. J. Soil Sci.* 85 67–74.

[B2] Ao Y. Sun M. Li Y. (2008). Effect of organic substrates on available elemental contents in nutrient solution. *Bioresour. Technol.* 99 5006–5010. 10.1016/j.biortech.2007.09.011 17967534

[B3] Barker A. V. Mills H. A. (1980). “Ammonium and nitrate nutrition of horticultural crops,” in *Horticultural Reviews*, ed. Janick J. (Hoboken, NJ: Wiley), 395–423.

[B4] Benoit F. Ceustermans N. (1995). *A Decade of Research on Ecologically Sound Substrates.* Leuven: International Society for Horticultural Science (ISHS), 17–30.

[B5] Birt D. F. Hendrich S. Wang W. (2001). Dietary agents in cancer prevention: flavonoids and isoflavonoids. *Pharmacol. Ther.* 90 157–177. 10.1016/S0163-7258(01)00137-1 11578656

[B6] Blackmer T. M. Schepers J. S. (1995). Use of a chlorophyll meter to monitor nitrogen status and schedule fertigation for corn. *J. Prod. Agric.* 8 56–60. 10.2134/jpa1995.0056

[B7] Blok C. Urrestarazu M. (2010). Substrate growing developments in Europe 2010-2027. *Horticom Plataforma.* Available online at: https://edepot.wur.nl/137023 (accessed September 1, 2020).

[B8] Børsheim K. Y. Christensen B. E. Painter T. J. (2001). Preservation of fish by embedment in Sphagnum moss, peat or holocellulose: experimental proof of the oxopolysaccharidic nature of the preservative substance and of its antimicrobial and tanning action. *Innov. Food Sci. Emerg. Technol.* 2 63–74. 10.1016/S1466-8564(00)00029-1

[B9] Claussen W. (2002). Growth, water use efficiency, and proline content of hydroponically grown tomato plants as affected by nitrogen source and nutrient concentration. *Plant Soil* 247 199–209. 10.1023/A:1021453432329

[B10] Clymo R. (1967). “Control of cation concentrations, and in particular of pH, in Sphagnum dominated communities,” in *Chemical Environment in the Aquatic Habitat*, eds Golterman H. L. Clymo R. S. (Amsterdam: North Holland), 273–284.

[B11] Clymo R. S. (1964). The origin of acidity in sphagnum bogs. *Bryologist* 67 427–431. 10.2307/3240768

[B12] R Core Team (2020). *R: A Language and Environment for Statistical Computing.* Vienna: R Foundation for Statistical Computing.

[B13] Dannehl D. Suhl J. Ulrichs C. Schmidt U. (2015). Evaluation of substitutes for rock wool as growing substrate for hydroponic tomato production. *J. Appl. Bot. Food Q.* 88 68–77. 10.5073/JABFQ.2015.088.010

[B14] De Boodt M. Verdonck O. (1972). “The physical properties of the substrates in horticulture,” in *III Symposium on Peat in Horticulture 26*, ed. Penningsfeld F. (Leuven: International Society for Horticultural Science (ISHS)), 37–44.

[B15] de Mendiburu F. (2020). *agricolae: Statistical Procedures for Agricultural Research. R package version 1.3-2.* Boston, MA: R Studio.

[B16] Domeño I. Irigoyen N. Muro J. (2009). Evolution of organic matter and drainages in wood fibre and coconut fibre substrates. *Sci. Hortic.* 122 269–274. 10.1016/j.scienta.2009.05.006

[B17] Economakis C. D. (1991). *Effect of Solution Conductivity on Growth and Yield of Lettuce in Nutrient Film Culture.* Leuven: International Society for Horticultural Science (ISHS), 309–316.

[B18] Encarnación C. Juan A. F. Diana N. Catalina E.-G. (2015). Nutrient solution aeration and growing cycles affect quality and yield of fresh-cut baby leaf red lettuce. *Agric. Food Sci.* 24 313–322. 10.23986/afsci.52792

[B19] Förster N. Ulrichs C. Schreiner M. Arndt N. Schmidt R. Mewis I. (2015). Ecotype variability in growth and secondary metabolite profile in Moringa oleifera: impact of sulfur and water availability. *J. Agric. Food Chem.* 63 2852–2861. 10.1021/jf506174v 25689922

[B20] Gruda N. (2009). Do soilless culture systems have an influence on product quality of vegetables? *J. Appl. Bot. Food Q.* 82 141–147.

[B21] Gruda N. Schnitzler W. H. (2004). Suitability of wood fiber substrate for production of vegetable transplants: I. Physical properties of wood fiber substrates. *Sci. Hortic.* 100 309–322. 10.1016/j.scienta.2003.10.001

[B22] Islam S. Khan S. Ito T. Maruo T. Shinohara Y. (2002). Characterization of the physico-chemical properties of environmentally friendly organic substrates in relation to rockwool. *J. Hortic. Sci. Biotechnol.* 77 143–148. 10.1080/14620316.2002.11511470

[B23] Jansson S. L. (1958). Tracer studies on nitrogen transformations in soil with special attention to mineralization-immobilization relationships. *Ann. Roy. Agr. Coll. Sweden* 24 101–361.

[B24] Kim M. J. Moon Y. Tou J. C. Mou B. Waterland N. L. (2016). Nutritional value, bioactive compounds and health benefits of lettuce (*Lactuca sativa* L.). *J. Food Compos. Anal.* 49 19–34. 10.1016/j.jfca.2016.03.004

[B25] Knight S. L. Mitchell C. A. (1983). Enhancement of lettuce yield by manipulation of light and nitrogen nutrition. *HortScience* 108 750–754. 11542284

[B26] Llorach R. Martínez-Sánchez A. Tomás-Barberán F. A. Gil M. I. Ferreres F. (2008). Characterisation of polyphenols and antioxidant properties of five lettuce varieties and escarole. *Food Chem.* 108 1028–1038. 10.1016/j.foodchem.2007.11.032 26065768

[B27] Massantini F. Favilli R. Magnani G. Oggiano N. (1988). Soilless culture, biotechnology for high quality vegetables. *Soilless Culture* 4 27–40.

[B28] McCall D. Willumsen J. (1998). Effects of nitrate, ammonium and chloride application on the yield and nitrate content of soil-grown lettuce. *J. Hortic. Sci. Biotechnol.* 73 698–703. 10.1080/14620316.1998.11511036

[B29] Müller I. Schmid B. Weiner J. (2000). The effect of nutrient availability on biomass allocation patterns in 27 species of herbaceous plants. *Perspect. Plant Ecol. Evol. Syst.* 3 115–127.

[B30] Nicolle C. Cardinault N. Gueux E. Jaffrelo L. Rock E. Mazur A. (2004). Health effect of vegetable-based diet: lettuce consumption improves cholesterol metabolism and antioxidant status in the rat. *Clin. Nutr.* 23 605–614. 10.1016/j.clnu.2003.10.009 15297097

[B31] Politycka B. Wójcik-Wojtkowiak D. Pudelski T. (1985). *Phenolic Compounds as A Cause of Phytotoxicity in Greenhouse Substrates Repeatedly used in Cucumber Growing.* Leuven: International Society for Horticultural Science (ISHS), 89–94.

[B32] Qadir O. Siervo M. Seal C. J. Brandt K. (2017). Manipulation of contents of nitrate, phenolic acids, chlorophylls, and carotenoids in lettuce (*Lactuca sativa* L.) via contrasting responses to nitrogen fertilizer when grown in a controlled Environment. *J. Agric. Food Chem.* 65 10003–10010. 10.1021/acs.jafc.7b03675 29059519

[B33] Quantis (2012). *Comparative Life Cycle Assessment of Horticultural Growing Media Based on Peat and Other Growing Media Constituents. Report prepared for the European Peat and Growing Media Association.* Lausanne: Quantis.

[B34] Rouphael Y. Cardarelli M. Lucini L. Rea E. Colla G. (2012). Nutrient solution concentration affects growth, mineral composition, phenolic acids, and flavonoids in leaves of artichoke and cardoon. *HortSci. Horts* 47:1424 10.21273/hortsci.47.10.1424

[B35] Rousk J. Brookes P. C. Bååth E. (2009). Contrasting soil pH effects on fungal and bacterial growth suggest functional redundancy in carbon mineralization. *Appl. Environ. Microbiol.* 75 1589–1596. 10.1128/AEM.02775-08 19151179 PMC2655475

[B36] Sahin U. Anapali O. Ercisli S. (2002). Physico-chemical and physical properties of some substrates used in horticulture. *Gartenbauwissenschaft* 67 55–60.

[B37] Santamaria P. Elia A. (1997). Producing nitrate-free endive heads: effect of nitrogen form on growth, yield, and ion composition of endive. *Am. Soc. Hortic. Sci.* 122 140–145. 10.21273/jashs.122.1.140

[B38] Shaw N. L. Cantliffe D. J. Funes J. Shine C. (2004). Successful Beit Alpha cucumber production in the greenhouse using pine bark as an alternative soilless media. *Am. Soc. Hortic. Sci.* 14 289–294. 10.21273/horttech.14.2.0289

[B39] Sonneveld C. (1991). “Rockwool as a substrate for greenhouse crops,” in *High-Tech and Micropropagation I*, ed. Bajaj Y. P. S. (Berlin: Springer), 285–312.

[B40] Veihmeyer F. J. Hendrickson A. H. (1931). The moisture equivalent as a measure of the field capacity of soils. *Soil Sci.* 32 181–194.

[B41] Wang J. Zhou Y. Zhou C. Shen Q. Putheti R. (2009). Effects of NH_4_^+^-N/NO_3_^–^-N ratios on growth, nitrate uptake and organic acid levels of spinach (Spinacia oleracea L.). *Afr. J. of Biotechnol.* 8 3597–3602. 10.5897/AJB2009.000-9356

[B42] Wilson G. C. S. (1984). *Tomato Production in Bark Substrates.* Leuven: International Society for Horticultural Science (ISHS), 271–276.

[B43] Xiong J. Tian Y. Wang J. Liu W. Chen Q. (2017). Comparison of coconut coir, rockwool, and peat cultivations for tomato production: nutrient balance, plant growth and fruit quality. *Front. Plant Sci.* 8:1327. 10.3389/fpls.2017.01327 28824665 PMC5539188

[B44] Xu H.-l Gauthier L. Gosselin A. (1995). Effects of fertigation management on growth and photosynthesis of tomato plants grown in peat, rockwool and NFT. *Sci. Hortic.* 63 11–20. 10.1016/0304-4238(95)00791-Q

[B45] Zhou W. Chen Y. Xu H. Liang X. Hu Y. Jin C. (2018). Short-term nitrate limitation prior to harvest improves phenolic compound accumulation in hydroponic-cultivated lettuce (*Lactuca sativa* L.) without reducing shoot fresh weight. *J. Agric. Food Chem.* 66 10353–10361. 10.1021/acs.jafc.8b02157 30222346

[B46] Zhou W. Liang X. Dai P. Chen Y. Zhang Y. Zhang M. (2019). Alteration of phenolic composition in lettuce (*Lactuca sativa* L.) by reducing nitrogen supply enhances its anti-proliferative effects on colorectal cancer cells. *Int. J. Mol. Sci.* 20:4205. 10.3390/ijms20174205 31466217 PMC6747510

